# Draft Genome Sequences of *Porphyromonas crevioricanis* JCM 15906^T^ and *Porphyromonas cansulci* JCM 13913^T^ Isolated from a Canine Oral Cavity

**DOI:** 10.1128/genomeA.00483-13

**Published:** 2013-07-25

**Authors:** Mitsuo Sakamoto, Naoto Tanaka, Yuh Shiwa, Hirofumi Yoshikawa, Moriya Ohkuma

**Affiliations:** Microbe Division/Japan Collection of Microorganisms RIKEN BioResource Center, Ibaraki, Japana; NODAI Culture Collection Center, Tokyo University of Agriculture, Tokyo, Japanb; Genome Research Center, NODAI Research Institute, Tokyo University of Agriculture, Tokyo, Japanc; Department of Bioscience, Tokyo University of Agriculture, Tokyo, Japand

## Abstract

Here, we report the draft genome sequences of *Porphyromonas crevioricanis* JCM 15906^T^ and *Porphyromonas cansulci* JCM 13913^T^, which were isolated from a canine oral cavity and were recently united under the single species *P. crevioricanis*. These two genome sequences are very similar, and yet a high degree of genome rearrangements is observed.

## GENOME ANNOUNCEMENT

*Porphyromonas crevioricanis* (1) and *Porphyromonas cansulci* (2) have been isolated from canine oral cavities. *P. crevioricanis* is one of the predominant bacterial species in subgingival plaque in dogs (3). It has been reported that *P. crevioricanis* strain JCM 15906^T^ shows a very high *hsp60* gene sequence similarity (100%) with that of *P. cansulci* JCM 13913^T^, as well as a very high 16S rRNA gene sequence similarity (99.9%) (4). Recently, *P. crevioricanis* and *P. cansulci* were found to be a single species, *P. crevioricanis*, based on the relatedness of their DNA (>91%) (5). Therefore, the genome sequences of two strains are expected to provide new insights into this species concept.

Chromosomal DNA was extracted from *P. crevioricanis* JCM 15906^T^ and *P. cansulci* JCM 13913^T^ using a Genomic-tip 100/G (Qiagen). Whole-genome sequencing was performed using an Illumina genome analyzer IIx, which produced paired-end reads of 101 bp with an insert size of 500 bp. *De novo* assemblies were performed using Velvet v1.1.02 (6), with parameters optimized by the VelvetOptimiser (http://www.vicbioinformatics.com/software.velvetoptimiser.shtml), resulting in 118 contigs with an N_50_ of 49,631 bp for *P. crevioricanis* JCM 15906^T^, comprising 2,044,812 bp, with an average G+C content of 45.3%, and 89 contigs with an N_50_ of 69,079 bp for *P. cansulci* JCM 13913^T^, comprising 2,108,435 bp with an average G+C content of 45.4%. The draft genomes were annotated by the Rapid Annotations using Subsystems Technology (RAST) sever (7) using Glimmer3 as a gene finder (8). The *P. crevioricanis* JCM 15906^T^ genome contains 2,086 protein-coding sequences (CDSs), a gene density of 88.6%, an average coding size of 864 bp, three rRNAs, and 48 tRNA sequences. The *P. cansulci* JCM 13913^T^ genome contains 2,180 CDSs, a gene density of 88.2%, an average coding size of 850 bp, three rRNAs, and 51 tRNA sequences. According to the genome BLAST distance phylogeny (GBDP)-based DNA-DNA hybridization (DDH) prediction (9), the pair of *P. crevioricanis* JCM 15906^T^ and *P. cansulci* JCM 13913^T^ genome sequences showed a 96.5% DDH value calculated by the Genome-to-Genome Distance Calculator (GGDC) web server (GGDC 2.0; http://ggdc.dsmz.de/distcalc2.php). BLAST dot plot analysis comparison in the SEED viewer (http://www.theseed.org) indicated a high degree of genome rearrangements between *P. crevioricanis* JCM 15906^T^ and *P. cansulci* JCM 13913^T^ genome sequences. *P. cansulci* JCM 13913^T^ contains large numbers of conjugative transposon proteins, which are frequently associated with genomic rearrangements. This might have contributed to rearrangement of the genomic structure and led to the diversification of *P. crevioricanis*. Further genome analyses will improve our understanding of this species.

### Nucleotide sequence accession numbers.

The draft genome sequences of *P. crevioricanis* JCM 15906^T^ and *P. cansulci* JCM 13913^T^ have been deposited in DDBJ/EMBL/GenBank under the accession no. BAOU00000000 and BAOV00000000.
